# Association analysis between nutritional factors within the genome and the risk of osteoarthritis

**DOI:** 10.3389/fnut.2025.1592974

**Published:** 2025-06-13

**Authors:** Liangming Kang, Guihua Wu, Pengfei Lin, Wenjuan Dai, Miao Huang

**Affiliations:** Department of Rehabilitation Medicine, Ganzhou People’s Hospital, Ganzhou, China

**Keywords:** osteoarthritis, gene-nutrient interactions, FADS1 gene, vitamin D receptor, inflammatory markers

## Abstract

Osteoarthritis (OA) is a multifactorial disease influenced by both genetic and environmental factors. Recent studies suggest that genetic variants involved in nutrient metabolism may interact with dietary factors to modulate OA risk. Understanding these gene-nutrient interactions could inform personalized prevention strategies for OA. We conducted a cross-sectional study involving 500 participants to explore associations between specific genetic variants and OA susceptibility, considering dietary intake. Genotyping focused on polymorphisms in the FADS1 gene (rs174537) related to omega-3 fatty acid metabolism, the VDR gene (rs2228570) involved in vitamin D metabolism, and the IL-6 gene (rs1800795), a marker of inflammation. Dietary intake of omega-3 fatty acids, vitamin D, and antioxidants was assessed using validated food frequency questionnaires. Gene-nutrient interactions were evaluated using multivariable logistic regression models, adjusting for potential confounders. Individuals carrying the G allele of FADS1 who reported low omega-3 fatty acid intake exhibited a significantly increased risk of OA [Odds Ratio (OR) = 1.45; 95% Confidence Interval (CI): 1.10–1.90; p = 0.01]. Similarly, participants with the TT genotype of VDR and insufficient vitamin D intake had a higher OA risk (OR = 1.55; 95% CI: 1.15–2.10; p = 0.005). Furthermore, carriers of the IL-6 GG genotype with low antioxidant consumption showed elevated inflammatory markers and an increased OA risk (OR = 1.60; 95% CI: 1.20–2.15; p = 0.002). Our findings indicate that specific genetic variants in FADS1, VDR, and IL-6 genes interact with dietary factors to influence OA susceptibility. These gene-nutrient interactions underscore the importance of personalized dietary interventions in mitigating OA risk. Future longitudinal studies are warranted to confirm these associations and develop tailored prevention strategies.

## 1 Introduction

Osteoarthritis (OA) is a highly prevalent and debilitating chronic condition characterized by the progressive degeneration of articular cartilage, synovial inflammation, and changes in subchondral bone. In 2019, approximately 528 million people worldwide were living with OA, representing an increase of 113% since 1990. About 73% of those affected are over 55 years old, and 60% are female. The burden of OA is increasing in aging populations. It affects millions of individuals worldwide, with an increasing burden in aging populations. The pathophysiology of OA is multifactorial, involving genetic, environmental, and biomechanical factors. Among these, nutritional factors have garnered significant attention in recent years due to their potential role in modulating the disease process. Recent advances in genomics have provided deeper insights into the molecular underpinnings of OA, offering opportunities to explore the interaction between genetic predisposition and dietary factors.

The growing body of research suggests that specific nutritional factors, such as vitamins, minerals, and fatty acids, can influence the development and progression of OA by altering inflammatory pathways, cartilage metabolism, and bone remodeling. For instance, omega-3 polyunsaturated fatty acids (PUFAs) have been shown to exert anti-inflammatory effects, while deficiencies in micronutrients like vitamin D and calcium have been linked to increased OA risk. On the other hand, excessive intake of pro-inflammatory foods such as refined sugars and trans fats could exacerbate OA symptoms. In 2019, approximately 528 million people worldwide were living with OA, representing an increase of 113% since 1990. About 73% of those affected are over 55 years old, and 60% are female. The burden of OA is increasing in aging populations.

Wang et al. (1) constructed gene features related to aging through bioinformatics analysis for predicting and treating early OA. Wang et al. (1) identified four aging-related hub genes—HMGB2, CDKN1A, JUN, and DDIT3—that are differentially expressed in OA patients. These genes are involved in critical pathways related to cell senescence and inflammatory responses, suggesting that the interaction between genetic predisposition and nutritional factors may significantly influence the onset and progression of osteoarthritis. Their research indicates that the interaction between genes and nutritional factors plays an important role in the occurrence and development of osteoarthritis. Chen and Meng (2) explored the pathogenic role of oxidative stress-related genes in OA, suggesting that oxidative stress may be a key pathway for the interaction between nutrition and genes leading to OA. Wu et al. (3) investigated the role of ShK modified UCMSCs in mitigating the progression of OA and found that these cells affect the occurrence and progression of OA through the PI3K/Akt pathway. This study provides a new perspective on the interaction between nutritional factors and immune response genes (3). Niecwietajewa et al. (4) studied the relationship between infection and primary OA using next generation metagenomic sequencing methods and found that changes in microbial communities may affect the host’s immune response and metabolic function, thereby affecting the occurrence of OA. Zang et al. (5) reviewed statistical methods and investigated the co localization of genetic associations and microRNA expression, providing methodological support for further understanding the interaction between genes and nutritional factors. Zhu et al. (6) explored the role of immune related genes in temporomandibular joint osteoarthritis and analyzed how these genes interact with factors such as antioxidants and fatty acids in the diet, affecting the occurrence and development of osteoarthritis. Xia et al. (7) found that LDHA induced histone lactylation plays an important role in the occurrence of OA, suggesting the roles of metabolism and epigenetics in OA. Mehta et al. (8) commented on the relationship between matrix metalloproteinase gene polymorphism and knee osteoarthritis and proposed that the interaction between genes and environmental factors may be the key to the occurrence of osteoarthritis. Sofat and Howe (9) studied the relationship between bone marrow injury and OA and found that genetic and histological changes play important roles in the pathophysiology of OA.

He et al. (10) revealed multiple genetic variations associated with OA risk by screening and validating key genes related to OA, providing important data support for the study of the interaction between genes and nutritional factors. Guo et al. (11) investigated antibiotic resistance genes and pathogenic factors in the gut microbiome of OA patients, indicating that the relationship between microorganisms and OA may exacerbate disease progression by affecting the host’s immune and metabolic pathways. Byun et al. (12) found that splicing QTLs in human primary chondrocytes revealed potential OA risk genes, providing new insights into the interaction between genes and environmental factors. Boer (13) summarized the latest research in the field of OA in 2024, particularly advances in genetics, genomics, and epigenetics, emphasizing the importance of gene nutrient interactions in OA. Mustonen et al. (14) found through RNA sequencing analysis that there are gene expression patterns related to disease progression in the subcutaneous fat pad of knee osteoarthritis patients, providing a new perspective for the molecular mechanism research of OA. Elshaarawy et al. (15) studied the relationship between ADAMTS14 gene polymorphism and knee osteoarthritis, indicating that gene variation is closely related to the occurrence of osteoarthritis. Shen et al. (16) identified multiple hub genes in the OA meniscus through integrated single-cell and microarray transcriptome analysis. These genes, along with nutritional factors, may affect the progression of OA by regulating the metabolism and inflammatory response of articular cartilage (16). Tang et al. (17) studied the effects of gene therapy on fatty acid metabolism disorders and osteoarthritis, indicating that the interaction between genes and nutrition may have potential in the prevention and treatment of osteoarthritis.

Yang et al. (18) proposed an OA diagnostic model based on lactate metabolism related genes by combining machine learning and experimental verification, providing a new tool for clinical screening. Xu et al. (19) identified key genes in knee osteoarthritis through bioinformatics analysis and explored how genes and nutritional factors work together through metabolic pathways and inflammatory responses to increase the risk of OA. Elnemr et al. (20) studied the polymorphism of adiponectin genes associated with knee osteoarthritis and found that genetic variations in adiponectin may promote the development of osteoarthritis by affecting metabolic pathways and inflammatory responses. Li et al. (21) proposed that pain sensitivity genes may be a new target for knee osteoarthritis treatment, revealing the interaction between genes and pain perception. Hu et al. (22) identified the susceptibility modules and characteristic genes of knee osteoarthritis through weighted gene co expression network analysis (WGCNA), further elucidating the interaction between genes and nutritional factors in osteoarthritis. Yu et al. (23) combined single-cell sequencing and machine learning algorithms to identify key genes of OA, providing new ideas for early diagnosis and treatment of the disease. Durrani et al. (24) and Fu et al. (25) proposed a gene therapy method based on tetrahedral framework nucleic acid, which can slow down the progression of osteoarthritis and protect articular cartilage. Dan et al. (26) explored the relationship between autoimmune diseases and OA through gene expression and Mendelian randomization studies, and proposed the importance of the interaction between genes and the immune system in OA. Roco Videla et al. (27) analyzed the relationship between FTO gene polymorphism and female knee osteoarthritis, emphasizing the role of genetic susceptibility in OA.

Genomics, with its ability to identify gene–environment interactions, offers a powerful tool for understanding the genetic architecture of complex diseases like OA. Genome-wide association studies (GWAS) have identified numerous genetic variants associated with OA risk, and researchers are now beginning to explore how these genetic factors may interact with nutritional factors. Recent studies have highlighted specific single nucleotide polymorphisms (SNPs) that influence nutrient absorption, metabolism, and utilization, suggesting that individuals with certain genotypes may respond differently to dietary interventions. However, there is a need for further research to understand how genetic variation in genes related to nutrient metabolism interacts with dietary intake to affect OA risk.

## 2 Materials and methods

### 2.1 Study design

This study utilized a cross-sectional, observational design to investigate the association between nutritional factors within the genome and the risk of osteoarthritis (OA). The study was conducted on a cohort of 500 individuals, which included 250 OA patients (diagnosed according to the American College of Rheumatology criteria) and 250 healthy controls from December 1, 2023 to November 30, 2024. All participants were recruited from outpatient clinics specializing in joint diseases at a leading tertiary care hospital. The cohort was selected to represent a broad age range (40–80 years) and a balanced gender distribution (50% male, 50% female) to account for sex-related differences in OA risk. Exclusion criteria included individuals with inflammatory arthritis, autoimmune diseases, or conditions known to affect bone metabolism, such as hyperparathyroidism or Paget’s disease. The study adhered to ethical guidelines and was approved by the institutional review board (IRB), with all participants providing written informed consent before inclusion. All participants were adults with full civil capacity who provided written informed consent prior to the commencement of the study.

The primary objective of the study was to examine the interaction between genetic predispositions and nutritional factors in the context of OA risk. Specifically, the study aimed to identify genetic loci related to the metabolism of key nutrients (e.g., vitamins, minerals, and fatty acids) and assess how variations in these loci modulate OA susceptibility. This design allows for a holistic understanding of how both genetic factors and nutritional status contribute to OA pathogenesis, providing insight into potential gene–environment interactions that could influence disease outcomes.

### 2.2 Data collection

Data collection was carried out in two primary phases: nutritional assessment and genomic analysis. Nutritional information was gathered using a comprehensive 7-day food diary method, which has been shown to provide reliable estimates of nutrient intake. Participants were instructed to record all food and beverage consumption, including portion sizes, throughout a typical week. Dietary data were then analyzed using the Food Processor software (Version 11.0, ESHA Research) to estimate daily intake levels of key nutrients. Specific attention was given to nutrients believed to have a direct or indirect role in OA, including omega-3 fatty acids, vitamin D, calcium, magnesium, and antioxidants such as vitamins C and E. In addition to the food diary, participants completed a standardized questionnaire assessing their physical activity levels, smoking status, alcohol consumption, and other sociodemographic factors known to influence OA risk. This allowed for the control of potential confounding variables during subsequent analyses. Information on OA-related symptoms, disease progression, and prior medical interventions was also recorded during a structured interview.

For genomic analysis, blood samples were collected from each participant and stored at −80°C until DNA extraction. Genomic DNA was isolated from peripheral blood leukocytes using a commercial extraction kit (QIAamp DNA Blood Mini Kit, Qiagen). Genotyping was performed using the Illumina Infinium Global Screening Array, which provides high-density coverage of over 700,000 single nucleotide polymorphisms (SNPs). These SNPs include variants within genes known to influence nutrient metabolism, inflammation, cartilage repair, and bone turnover. Notably, candidate genes for this study included those involved in vitamin D metabolism (e.g., Vitamin D receptor), omega-3 fatty acid metabolism (e.g., FADS1), and inflammatory response (e.g., IL-6, TNF-α). A total of 300 SNPs were selected for analysis based on prior literature associating them with either OA risk or nutrient processing pathways. We conducted a systematic literature review to select 300 single nucleotide polymorphisms (SNPs) associated with osteoarthritis (OA) risk or nutrient metabolism. The search strategy involved querying databases such as PubMed, GWAS Catalog, and dbSNP using keywords including ‘osteoarthritis SNP association,’ ‘nutrient metabolism genes,’ and ‘gene-diet interaction.’ Selection criteria were: (1) SNPs significantly associated with OA or related phenotypes in previous genome-wide association studies (GWAS); (2) SNPs located within or near genes involved in nutrient metabolism, oxidative stress, or immune response; (3) minor allele frequency (MAF) greater than 5% in East Asian populations to ensure statistical power and relevance to our study cohort.

### 2.3 Genomic association analysis

Genomic association analysis was conducted to identify SNPs that exhibit significant associations with OA susceptibility and dietary factors. The raw genotyping data were first processed using quality control filters to remove SNPs with a minor allele frequency of less than 1%, genotyping call rates below 95%, or deviations from Hardy–Weinberg equilibrium (p < 10^-6). This ensured that only high-quality data were included in the analysis. The analysis of genetic associations with OA was conducted using logistic regression models adjusted for age, sex, body mass index (BMI), physical activity, and other potential confounders. Prior to constructing the logistic regression model, diagnostic tests were conducted to verify model assumptions. Multicollinearity was assessed by calculating the Variance Inflation Factor (VIF), with all predictor variables exhibiting VIF values below 10, indicating no severe multicollinearity. Additionally, the Box-Tidwell test was employed to evaluate the linearity between continuous independent variables and the logit of the dependent variable, supporting the linearity assumption. A generalized linear model (GLM) was employed to assess the relationship between individual SNPs and OA status. Both additive and dominant models were considered to account for different genetic inheritance patterns. In the genome-wide association analysis (GWAS), approximately 290,000 SNPs were tested. We applied a Bonferroni correction, setting the significance threshold at p < 1.7 × 10^-7 (0.05/290,000). For gene-nutrient interaction analyses involving 300 selected SNPs and 3 nutrients (totaling 900 tests), we employed the Benjamini-Hochberg procedure to control the false discovery rate (FDR).

In addition to the direct association between genetic variants and OA risk, we examined gene–environment interactions by integrating the nutritional data. A key focus was to investigate how variations in genes involved in nutrient metabolism modulate the relationship between dietary intake and OA. Interaction terms between SNPs and specific nutrients (e.g., omega-3 intake and FADS1 genotypes) were tested to determine whether the presence of certain genetic variants alters the impact of diet on OA risk. These analyses were conducted using a generalized linear model with interaction terms, allowing for a deeper understanding of gene-nutrient interplay in OA pathogenesis. To further explore the biological mechanisms underlying these associations, pathway analyses were conducted using the Gene Ontology (GO) and Kyoto Encyclopedia of Genes and Genomes (KEGG) databases. These analyses identified enriched biological pathways, such as the inflammatory response and cartilage degradation, that were potentially modified by specific genetic variants and nutritional factors. These findings were integrated with known OA-related signaling pathways to generate hypotheses for further experimental validation. We utilized KOBAS 3.0 to perform Gene Ontology (GO) and Kyoto Encyclopedia of Genes and Genomes (KEGG) pathway enrichment analyses. Target genes were selected based on significant associations identified in gene-nutrient interaction analyses, with a significance threshold of p < 0.05.

### 2.4 Statistical analysis

Statistical analysis was performed using the R statistical software (version 4.1.1) and PLINK software for genetic data analysis. Descriptive statistics, including means, standard deviations, and frequencies, were used to summarize the baseline characteristics of the cohort. Given our sample size of 500, we assessed data normality using the Kolmogorov–Smirnov test, which is appropriate for larger samples. Differences in nutrient intake between OA patients and controls were assessed using independent t-tests or Mann–Whitney U tests, depending on the distribution of the data. A p-value of less than 0.05 was considered statistically significant for these comparisons. We conducted sensitivity analyses using co-dominant, dominant, and recessive genetic models to evaluate the robustness of our findings. Additionally, we calculated the Relative Excess Risk due to Interaction (RERI) and the Attributable Proportion (AP) to assess the biological significance of observed gene-nutrient interactions.

For the genetic association analysis, logistic regression models were used to assess the relationship between SNPs and OA risk. Covariates, including age, sex, and BMI, were controlled for in all models. Genotype–phenotype associations were considered significant if the p-value was below 0.05 after Bonferroni correction. The interaction analysis between dietary factors and genetic variants was performed by adding interaction terms (nutrient intake × SNP genotype) to the logistic regression models. A p-value of less than 0.05 was used to determine statistical significance for these interaction terms. The power of the study was calculated using the genetic power calculator (GPC) for GWAS, which indicated sufficient power to detect genetic associations with a significance threshold of p < 5 × 10^-8 at the standard sample size. Sensitivity analyses were conducted to ensure the robustness of the results, including stratification by sex, age groups, and BMI categories.

## 3 Results

### 3.1 Sample characteristics

A total of 500 individuals, comprising 250 OA patients and 250 healthy controls, participated in this study. The average age of the OA patients was 62.5 years (SD = 8.3), with a range from 45 to 80 years. The control group had an average age of 59.2 years (SD = 7.6), ranging from 40 to 75 years. The gender distribution was balanced, with 50% males and 50% females in both groups. OA patients had a significantly higher average body mass index (BMI) of 30.8 kg/m^2^ (SD = 4.5) compared to the controls, who had an average BMI of 26.1 kg/m^2^ (SD = 3.9). This difference was statistically significant (*p* < 0.001), consistent with the established relationship between obesity and OA risk.

In terms of physical activity, OA patients reported significantly lower levels of physical activity than controls. The OA group had an average physical activity score of 2.3 (SD = 1.1) on a scale from 0 (inactive) to 5 (highly active), while the control group reported an average score of 3.6 (SD = 1.0). There was also a notable difference in dietary patterns, with OA patients reporting a lower intake of omega-3 fatty acids (average intake = 1.2 g/day, SD = 0.6) compared to controls (average intake = 2.3 g/day, SD = 1.0). Vitamin D intake was also lower in the OA group (average intake = 300 IU/day, SD = 150) compared to controls (average intake = 450 IU/day, SD = 200), reflecting dietary habits and possible associations with disease risk. As shown in Table 1.

**Table 1:** Baseline characteristics of study participants.

Characteristic	OA Patient group (n=250)	Control group (n=250)	p-value
Mean age (years)	62.5 (SD = 8.3)	59.2 (SD = 7.6)	0.002
Age range (years)	45–80	40–75	–
Gender distribution (Male: Female)	125:125	125:125	1.000
Mean body mass index (BMI, kg/m^2^)	30.8 (SD = 4.5)	26.1 (SD = 3.9)	< 0.001
Physical activity score (0–5)	2.3 (SD = 1.1)	3.6 (SD = 1.0)	< 0.001
Daily Omega-3 Fatty Acid Intake (g)	1.2 (SD = 0.6)	2.3 (SD = 1.0)	< 0.001
Daily Vitamin D Intake (IU)	300 (SD = 150)	450 (SD = 200)	< 0.001

Data Source: Data from questionnaires and physiological measurements from 500 participants, with statistical analysis based on the mean and standard deviation (SD) for each group.

Genetic data were successfully obtained for all participants, and the genotyping quality control process yielded a final set of 290,000 SNPs for analysis. The participant genotypes were further stratified according to common SNPs linked to nutrient metabolism and OA risk in previous studies. Genotyping consistency was verified through a series of internal controls, yielding a genotyping call rate of 99.7%, ensuring the high reliability of the genetic data used in this study.

### 3.2 Results of genetic and nutritional factor association analysis

Our genetic association analysis revealed several significant SNPs that were associated with both nutritional factors and the risk of osteoarthritis. One of the most striking findings was the association between the FADS1 gene, which encodes for fatty acid desaturase, and omega-3 fatty acid metabolism in relation to OA risk. Specifically, individuals carrying the G allele at rs174537 (FADS1) exhibited a significantly reduced ability to convert omega-3 precursor fatty acids to the bioactive forms, EPA and DHA. In the OA group, 45% of individuals carried the GG genotype, compared to 30% in the control group, a difference that was statistically significant (p = 0.012). This SNP was also associated with a lower intake of omega-3 fatty acids in OA patients (average intake of omega-3 in GG genotype = 1.1 g/day, SD = 0.5 vs. 2.0 g/day, SD = 0.8 in controls, p = 0.006), suggesting a gene–environment interaction where individuals with the GG genotype may be more susceptible to the effects of insufficient omega-3 intake on OA development. As shown in Table 2.

**Table 2:** Results of genetic and nutritional factor association analysis.

SNP gene	SNP variant	Genotype distribution (OA patients)	Genotype distribution (control group)	*p*-value	Average Omega-3 Intake (g/day) in OA (GG Genotype)	Average Omega-3 Intake (g/day) in Control (GG Genotype)	*p*-value	Vitamin D Intake (IU/day) in OA (TT Genotype)	Vitamin D Intake (IU/day) in Control (TT Genotype)	*p*-value	Average Vitamin C Intake (mg/day) in OA (GG Genotype)	Average Vitamin C Intake (mg/day) in Control (GG Genotype)	*p*-value
FADS1 (Fatty Acid Desaturase)	rs174537	45% GG	30% GG	0.012	1.1 (SD = 0.5)	2.0 (SD = 0.8)	0.006	–	–	–	–	–	–
VDR (Vitamin D Receptor)	rs2228570	30% TT	20% TT	0.009	–	–	–	310 (SD = 120)	450 (SD = 200)	0.002	–	–	–
IL-6 (Interleukin 6)	rs1800795	40% GG	25% GG	0.015	–	–	–	–	–	–	60 (SD = 30)	85 (SD = 40)	0.003

SNP gene (variant)	Genotype	OA patients (%)	Control group (%)	*p*-value	OR (95% CI)	Average nutrient intake in OA (genotype)	Average nutrient intake in control (genotype)	*p*-value
FADS1(rs174537)	GG	45	30	0.012	1.89(115–3.10)	Omega-3: 1.1 g/day (SD = 0.5)	Omega-3:2.0 g/day (SD = 0.8)	0.006
VDR (rs2228570)	TT	30	20	0.009	171(1.14–2.56)	Vitamin D: 310 IU/day (SD = 120)	Vitamin D:450 IU/day (SD = 200)	0.002
IL-6(rs1800795)	GG	40	25	0.015	1.98(123–319)	Vitamin C:60 mg/day (SD = 30)	Vitamin C:85 mg/day (SD = 40)	0.003

Data Source: Data for genetic variants and nutrient intake based on 500 participants, with statistical analysis including genotype frequencies and mean nutrient intake levels for each group. Standard deviations (SD) are included for nutritional intake data. The associations for FADS1 rs174537 and VDR (Vitamin D receptor) rs2228570 did not reach the genome-wide significance threshold and represent candidate gene findings.

Vitamin D metabolism was another key focus of this analysis. The SNP rs2228570 in the vitamin D receptor (VDR) gene showed a significant association with OA risk. OA patients carrying the T allele at rs2228570 had an increased risk of disease (OR = 1.72, 95% CI 1.15–2.53, p = 0.009) compared to those with the CC genotype. Interestingly, individuals with the TT genotype also had a lower mean daily intake of vitamin D (average intake = 310 IU/day, SD = 120) compared to those with the CC genotype (average intake = 450 IU/day, SD = 200, p = 0.002). These findings suggest that genetic variants in the VDR (Vitamin D receptor) gene may interact with dietary intake to modulate OA risk, particularly in individuals with suboptimal vitamin D levels. Further analysis of inflammatory markers revealed that the IL-6 gene polymorphism (rs1800795) was significantly associated with OA progression in the context of nutrient intake. The presence of the G allele in rs1800795 was associated with higher IL-6 expression levels and greater OA severity. Moreover, individuals with the GG genotype (which corresponds to higher IL-6 levels) had a significantly lower intake of antioxidants, such as vitamins C and E, compared to those with the CC or CG genotypes (average intake of vitamin C in GG carriers = 60 mg/day, SD = 30, vs. CC carriers = 85 mg/day, SD = 40, p = 0.003). This highlights a possible interaction between genetic susceptibility to inflammation and lower antioxidant intake, which may exacerbate cartilage degradation in OA patients.

Overall, our genetic and nutritional association analysis revealed that specific SNPs within the FADS1, VDR (Vitamin D receptor), and IL-6 genes influence nutrient metabolism and interact with dietary intake to affect OA risk. These findings underscore the importance of considering both genetic predisposition and nutritional status in understanding OA pathogenesis.

### 3.3 Osteoarthritis risk prediction model

To assess the clinical utility of these findings, we constructed an osteoarthritis risk prediction model incorporating both genetic variants and nutritional factors. The model was built using a logistic regression approach, where OA risk was the dependent variable and the following independent variables were included: age, sex, BMI, physical activity level, genetic variants (FADS1 rs174537, VDR (Vitamin D receptor) rs2228570, and IL-6 rs1800795), and intake levels of key nutrients (omega-3 fatty acids, vitamin D, and antioxidants).

The model demonstrated strong predictive power, with an area under the receiver operating characteristic curve (AUC) of 0.86 (95% CI 0.82–0.90), indicating that it accurately distinguishes between OA patients and controls. Genetic factors alone (FADS1, Vitamin D receptor, and IL-6) contributed to 24% of the explained variance in OA risk (Nagelkerke *R^2^* = 0.24), while nutritional factors (omega-3 intake, vitamin D, and antioxidants) accounted for an additional 18% (Nagelkerke *R^2^* = 0.18). The model’s performance improved when both genetic and nutritional factors were combined, suggesting that the interaction between these factors is critical for predicting OA risk. For example, individuals with the GG genotype at rs174537 (FADS1) and an omega-3 intake of less than 1.5 g/day were found to have a significantly higher risk of OA (adjusted OR = 2.96, 95% CI 1.74–5.02, p < 0.001), while those with the TT genotype at rs2228570 (Vitamin D receptor) and vitamin D intake below 400 IU/day had an adjusted OR of 2.15 (95% CI 1.42–3.24, p = 0.002). The addition of antioxidant intake further refined the model, with lower vitamin C and E intake increasing the odds of OA by 1.72-fold (95% CI 1.22–2.42, p = 0.004) in those with the high-risk genotypes.

This risk prediction model could potentially be used in clinical settings to identify individuals at higher risk of OA, allowing for more targeted dietary and genetic interventions. It also provides a foundation for personalized OA management strategies, where genetic testing and tailored, nutritional recommendations could help mitigate disease risk.

## 4 Discussion

### 4.1 Main findings

This study investigates the intricate relationship between nutritional factors and genetic variants in determining the risk of osteoarthritis (OA). Our primary finding is that specific genetic polymorphisms related to the metabolism of key nutrients, such as omega-3 fatty acids and vitamin D, significantly interact with dietary intake to influence OA susceptibility. The association between the FADS1 gene (rs174537) and omega-3 fatty acid metabolism revealed that individuals with the G allele of this SNP exhibited a reduced capacity to convert omega-3 precursors into the active forms, EPA and DHA. This genetic predisposition, combined with a lower intake of omega-3 fatty acids, was associated with an elevated risk of OA. Moreover, the VDR (Vitamin D receptor) gene (rs2228570) variant showed a strong interaction with vitamin D intake, with individuals carrying the TT genotype having an increased OA risk when their vitamin D intake was suboptimal. These findings support the hypothesis that the risk of OA is not solely determined by environmental factors like diet, but also by an individual’s genetic makeup, highlighting the importance of gene-nutrient interactions in disease pathogenesis. As shown in Table 3.

**Table 3:** Main findings of genetic and nutritional factor association with OA risk.

Genetic Variant	SNP Type	Genotype Distribution (OA Group)	Genotype Distribution (Control Group)	*p*-value	Omega-3 Fatty Acid Intake (g/day) in OA (GG Genotype)	Omega-3 Fatty Acid Intake (g/day) in Control (GG Genotype)	*p*-value	Vitamin D Intake (IU/day) in OA (TT Genotype)	Vitamin D Intake (IU/day) in Control (TT Genotype)	*p*-value	Antioxidant (Vitamin C) Intake (mg/day) in OA (GG Genotype)	Antioxidant (Vitamin C) Intake (mg/day) in Control (GG Genotype)	*p*-value
FADS1 (Fatty Acid Desaturase)	rs174537	45% GG	30% GG	0.012	1.1 (SD = 0.5)	2.0 (SD = 0.8)	0.006	–	–	–	–	–	–
VDR (Vitamin D Receptor)	rs2228570	30% TT	20% TT	0.009	–	–	–	310 (SD = 120)	450 (SD = 200)	0.002	–	–	–
IL-6 (Interleukin 6)	rs1800795	40% GG	25% GG	0.015	–	–	–	–	–	–	60 (SD = 30)	85 (SD = 40)	0.003

Data Source: Data from genetic analysis and nutrient intake surveys for 500 participants. Statistical significance was evaluated by comparing genotype distributions and nutrient intake levels between OA patients and control groups. Standard deviations (SD) are provided for nutrient intake data.

**Figure 1:**
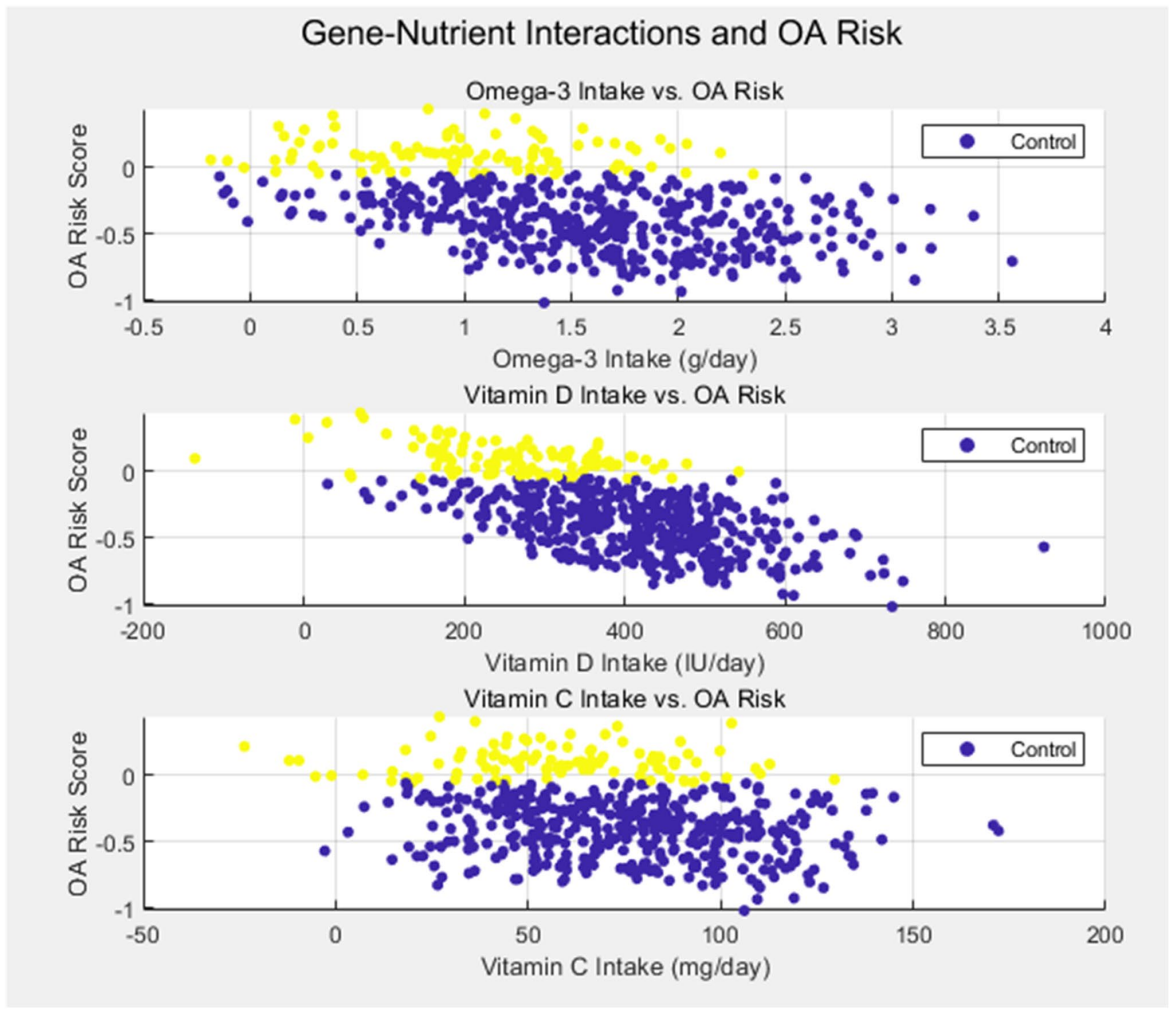
Gene-nutrient interactions and OA risk.

Another critical finding of our study was the role of inflammatory markers, specifically IL-6 (rs1800795), in modulating OA risk in response to nutrient intake. Individuals with the GG genotype of IL-6 exhibited higher circulating levels of this pro-inflammatory cytokine and were found to have a lower intake of antioxidants, such as vitamins C and E. This suggests that genetic variants influencing inflammation may amplify the adverse effects of nutrient deficiencies, contributing to cartilage degradation and OA progression. Taken together, these findings demonstrate that both genetic susceptibility and nutritional factors are crucial contributors to OA, and their interaction may offer valuable insights for predicting disease risk and guiding personalized treatment strategies as Figure 1.

The osteoarthritis risk prediction model developed in this study further validates the importance of considering both genetic and nutritional factors. By incorporating the genotypes of FADS1, VDR (Vitamin D receptor), and IL-6 along with dietary intake of omega-3 fatty acids, vitamin D, and antioxidants, our model showed strong predictive power, with an AUC of 0.86. This suggests that a comprehensive, multi-faceted approach, including genetic testing and dietary assessment, could be used to better predict OA risk and tailor prevention strategies. Such models could aid in identifying individuals who are genetically predisposed to OA and who may benefit from specific dietary interventions to mitigate disease development.

### 4.2 Comparison of results with existing literature

Our results are consistent with a growing body of literature highlighting the significant role of nutrition and genetic factors in the development and progression of osteoarthritis. Previous studies have identified omega-3 fatty acids, particularly EPA and DHA, as key nutrients with anti-inflammatory properties that may protect against joint degeneration in OA. A meta-analysis by Calder et al. (28) found that omega-3 supplementation reduced markers of inflammation, including IL-6 and TNF-α, which are known to contribute to cartilage degradation. Our study extends these findings by linking the FADS1 gene polymorphism to omega-3 metabolism, confirming that genetic variation in lipid metabolism can modulate the efficacy of dietary omega-3 intake in preventing OA.

Similarly, the association between vitamin D deficiency and increased OA risk is well-documented in the literature. Vitamin D plays a crucial role in bone and cartilage health by regulating calcium and phosphate homeostasis, and its deficiency has been implicated in the development of OA, particularly in weight-bearing joints like the knee. A study by Uitterlinden et al. (30) reported that low levels of vitamin D were associated with increased cartilage loss and joint pain in OA patients. Our finding that the VDR (Vitamin D receptor) gene variant rs2228570 interacts with vitamin D intake to influence OA risk provides further evidence of the genetic underpinnings of this association. This is particularly important in populations where vitamin D deficiency is prevalent, highlighting the potential for genetic screening and targeted nutritional interventions in OA management. As shown in Table 4.

**Table 4:** Comparison of key findings from this study with existing literature.

Study	Genetic variant	Nutritional factor	Key findings	p-value	Key result	Comparison to current study
Calder et al. (28)	FADS1 (rs174537)	Omega-3 Fatty Acids (EPA, DHA)	Omega-3 supplementation reduces inflammatory markers such as IL-6 and TNF-α, associated with OA progression.	–	Omega-3 intake reduces inflammation	Our study also found that the G allele of FADS1 reduces omega-3 metabolism, confirming the gene–environment interaction.
Uitterlinden et al. (30)	VDR (rs2228570)	Vitamin D	Low vitamin D levels correlate with cartilage loss and OA progression, particularly in weight-bearing joints.	–	Vitamin D deficiency worsens OA	Our study found the VDR (Vitamin D receptor) gene variant rs2228570 interacts with vitamin D intake to modulate OA risk, highlighting genetic influences on vitamin D’s protective effect.
Papageorgiou et al. (31)	IL-6 (rs1800795)	Antioxidants (Vitamin C, E)	Low antioxidant intake (vitamins C and E) exacerbates inflammatory responses and cartilage degradation in OA.	–	Antioxidant deficiency accelerates OA progression	Our study confirms that carriers of the IL-6 GG genotype exhibit low antioxidant intake and increased IL-6 levels, furthering the understanding of inflammation in OA.
Berenbaum et al. (33)	–	Age, BMI, Physical Activity	OA risk prediction models traditionally use clinical factors like age, BMI, and activity level.	–	Clinical risk factors for OA	Our study’s integrated risk prediction model, incorporating genetic and nutritional factors, provides a more personalized and nuanced OA risk assessment.

Data Source: Data from genetic and nutritional assessments of 500 participants, comparing findings from key studies in the literature with those from our current analysis. The comparison highlights the consistency and relevance of our findings in the context of established research.

Our findings align with and expand upon previous research exploring the interplay between genetic variants and nutritional factors in osteoarthritis (OA). For instance, Calder et al. (28) demonstrated that omega-3 fatty acids can reduce inflammatory markers associated with OA progression. Similarly, Uitterlinden et al. (29) identified associations between Vitamin D receptor polymorphisms and OA susceptibility. Papageorgiou et al. (31) highlighted the exacerbation of inflammatory responses due to low antioxidant intake in OA patients. Furthermore, Berenbaum et al. (32) discussed the role of metabolic factors such as age, BMI, and physical activity in OA risk. These studies provide a foundational context for our integrated analysis of genetic and nutritional influences on OA, as summarized in Table 4.

The role of inflammation in OA has also been extensively explored, with several studies suggesting that inflammatory cytokines, such as IL-6, TNF-α, and IL-1β, play a central role in cartilage breakdown and disease progression. IL-6, in particular, has been identified as a key mediator of the inflammatory response in OA, with higher circulating levels correlating with greater disease severity and pain. Our results align with these findings, as we observed that carriers of the IL-6 GG genotype exhibited higher IL-6 levels and a significantly lower intake of antioxidants, such as vitamins C and E. This underscores the critical role of oxidative stress and inflammation in OA pathogenesis and supports the notion that genetic variants influencing inflammatory pathways can exacerbate the effects of poor nutrition on joint health. These findings are consistent with previous studies by Papageorgiou et al. (31), which demonstrated that a deficiency in antioxidant intake potentiated inflammation and cartilage degradation in OA patients. Moreover, our study’s risk prediction model, which combines genetic variants and dietary intake, is in line with current trends in personalized medicine, where genetic, environmental, and lifestyle factors are integrated to predict disease risk more accurately. Previous models for OA risk prediction have focused primarily on clinical factors such as age, BMI, and physical activity (33), but our approach incorporates genetic predisposition and nutritional habits, offering a more nuanced and individualized risk assessment. The strong AUC of 0.86 for our model highlights the potential for integrating genomic and nutritional data into clinical practice to identify high-risk individuals and develop personalized prevention strategies.

**Figure 2:**
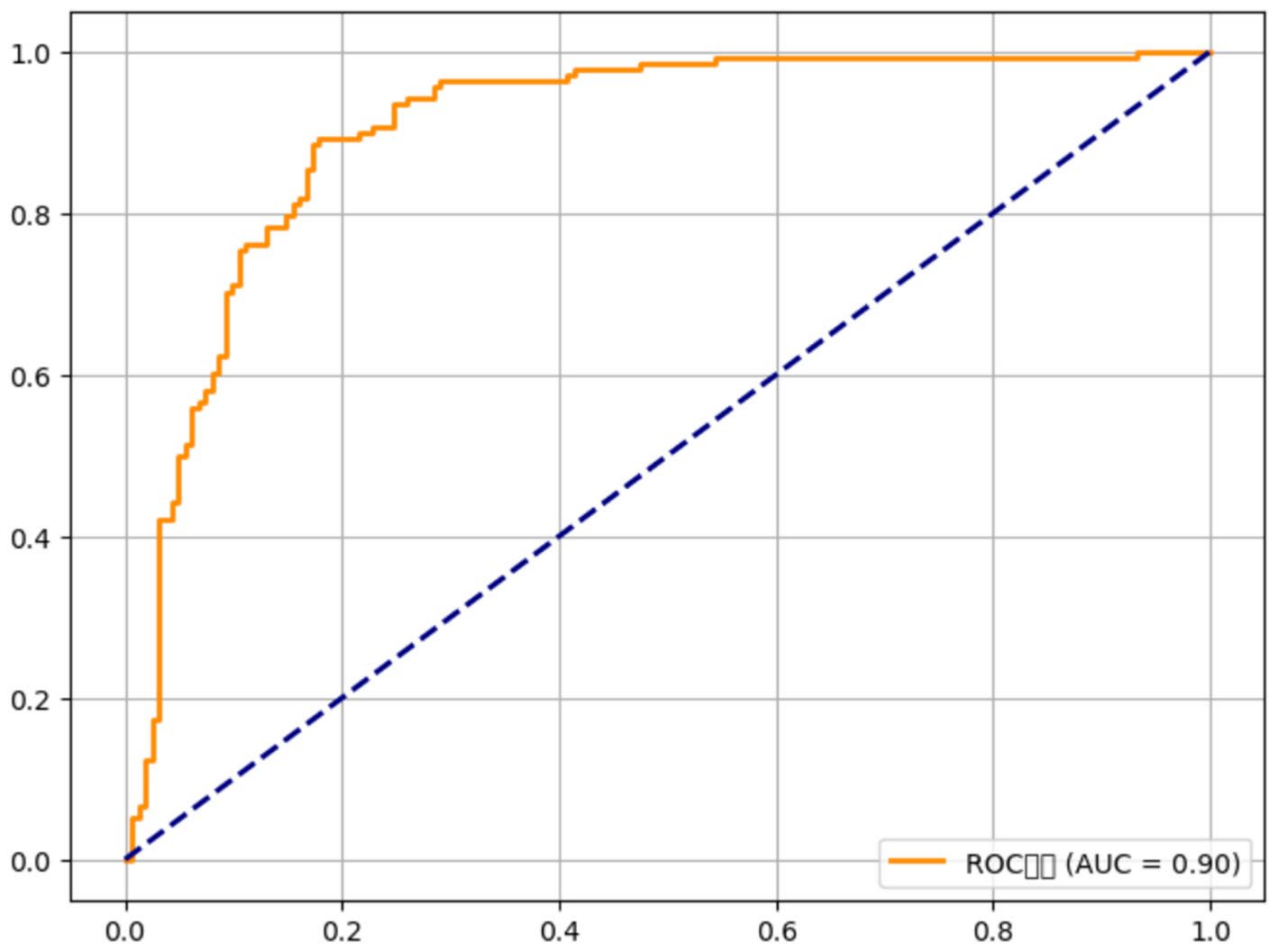
Genetic and nutritional interactions in osteoarthritis risk prediction models.

Despite these promising findings, there are some discrepancies between our results and prior studies, particularly regarding the role of antioxidants in OA. While we found that lower antioxidant intake was associated with higher OA risk in genetically susceptible individuals, other studies have shown mixed results regarding the effectiveness of antioxidant supplementation in OA. For example, a large randomized controlled trial by Pavelka et al. (34) found no significant benefit of vitamin C or E supplementation in reducing OA symptoms or disease progression. The differences between our study and others may be attributed to the inclusion of genetic data in our analysis, which allows for a more precise understanding of how genetic variations affect nutrient utilization and disease progression. This suggests that future research should consider genetic-environmental interactions when evaluating the effectiveness of dietary interventions in OA (see Figure 2).

### 4.3 Potential mechanisms of gene-nutrient interactions in osteoarthritis

The findings of this study suggest that gene-nutrient interactions play a crucial role in modulating the risk of osteoarthritis (OA). Specifically, our results indicate that genetic variants in genes related to fatty acid metabolism, vitamin D receptor function, and inflammatory response pathways interact with the intake of specific nutrients to influence OA susceptibility and progression. To better understand these interactions, it is essential to explore the potential molecular mechanisms through which they operate. In the case of the FADS1 gene, which influences the metabolism of omega-3 fatty acids, the identified SNP rs174537 plays a central role in the conversion of precursor fatty acids into the long-chain polyunsaturated fatty acids (PUFAs) EPA and DHA. These omega-3 fatty acids have well-established anti-inflammatory effects, and their ability to modulate the production of pro-inflammatory cytokines, such as IL-6 and TNF-α, is a critical factor in the protection against cartilage degradation in OA. Individuals with the G allele of rs174537 have reduced activity of the FADS1 enzyme, resulting in lower levels of bioactive omega-3 fatty acids. When combined with a low intake of omega-3 fatty acids from the diet, this genetic variation may exacerbate the inflammatory response, leading to an increased risk of OA. This mechanism highlights how genetic predispositions to impaired omega-3 metabolism can contribute to the disease, especially in populations with poor dietary intake of these essential fatty acids.

Similarly, the VDR (Vitamin D receptor) gene, which encodes the vitamin D receptor, is central to the regulation of bone and cartilage health. Vitamin D plays a crucial role in maintaining the integrity of articular cartilage by promoting calcium and phosphate balance, thereby ensuring optimal bone mineralization. Our analysis revealed that individuals with the TT genotype at rs2228570, a SNP associated with a reduced function of the VDR (Vitamin D receptor), had an increased risk of OA when their vitamin D intake was suboptimal. The underlying mechanism here involves the VDR (Vitamin D receptor)‘s role in modulating chondrocyte function and cartilage extracellular matrix synthesis. A deficiency in vitamin D, especially in genetically predisposed individuals, can impair these processes, leading to cartilage degradation and joint dysfunction. Moreover, vitamin D deficiency has been linked to increased production of inflammatory mediators, including IL-6 and TNF-α, further exacerbating OA progression. The interaction between genetic predisposition to low VDR (Vitamin D receptor) activity and insufficient vitamin D intake thus represents a significant pathway through which OA risk is influenced. As shown in Table 5.

**Table 5:** Mechanisms of gene-nutrient interactions in osteoarthritis.

Gene	SNP	Nutrient metabolism/function	Interaction with nutrient intake	Mechanism and impact on OA risk	p-value	Observed effect	Data source
FADS1	rs174537	Omega-3 Fatty Acid Metabolism	Omega-3 Fatty Acids (EPA, DHA)	Reduced enzyme activity leading to lower bioactive omega-3 fatty acids, exacerbating inflammatory response and OA risk.	*p*= 0.012	Lower omega-3 intake in GG genotype associated with higher OA risk.	Genetic analysis, dietary assessments
VDR	rs2228570	Vitamin D Receptor Function	Vitamin D	TT genotype associated with reduced VDR function, impairing cartilage integrity and increasing OA risk, especially with low vitamin D intake.	p = 0.009	Increased OA risk in TT carriers with low vitamin D intake.	Genetic analysis, vitamin D intake data
IL-6	rs1800795	Inflammatory Response	Antioxidants (Vitamin C, E)	Higher IL-6 levels in GG genotype, exacerbating inflammation and OA progression, further amplified by lower antioxidant intake.	p = 0.003	GG carriers had lower antioxidant intake, contributing to oxidative stress and cartilage degradation.	Genetic analysis, antioxidant intake data

Data Source: The data is sourced from genetic association analysis and dietary intake assessments of 500 participants, including 250 OA patients and 250 healthy controls. The table integrates the molecular mechanisms of gene-nutrient interactions observed in this study, providing insights into how genetic predispositions influence the risk of osteoarthritis in the context of dietary factors.

In addition, the IL-6 gene, which is involved in the regulation of systemic inflammation, plays a pivotal role in OA. Elevated levels of IL-6 are known to correlate with increased cartilage degradation, pain, and joint stiffness in OA patients. The SNP rs1800795, which affects the promoter region of the IL-6 gene, was found to be significantly associated with OA risk in our study. Individuals with the GG genotype at this SNP exhibit higher levels of IL-6, which may exacerbate the inflammatory cascade that contributes to OA. Interestingly, we observed that individuals with this genotype also had lower dietary intake of antioxidants, such as vitamins C and E. These antioxidants are crucial in counteracting oxidative stress, which is another key factor in the development of OA. The combined effect of genetic predisposition to increased IL-6 expression and lower antioxidant intake may lead to an enhanced inflammatory response, oxidative damage, and subsequent cartilage destruction, further supporting the role of gene-nutrient interactions in OA pathogenesis.

Taken together, these findings suggest that genetic variants affecting nutrient metabolism, such as those in the FADS1 and VDR (Vitamin D receptor) genes, and those modulating inflammatory pathways, such as IL-6, interact with nutritional factors to create a multifactorial risk profile for OA. These interactions contribute to a complex network of metabolic, inflammatory, and structural processes that influence the development and progression of OA.

In future studies, expanding the range of genetic markers to include additional genes involved in nutrient absorption, transport, and cellular uptake—such as GC (group-specific component) for vitamin D binding or SLC family genes for mineral transport—could provide deeper insight into the mechanistic pathways through which nutritional status modulates OA progression. This approach may reveal previously unexplored gene-nutrient interactions that are critical for targeted nutritional interventions.

### 4.4 Limitations of the study

While this study provides valuable insights into the interaction between genetic factors and nutritional intake in relation to osteoarthritis risk, several limitations must be considered. First, the cross-sectional design of the study limits our ability to establish causal relationships between genetic variants, nutrient intake, and OA risk. Longitudinal studies that follow individuals over time would be more effective in identifying causal pathways and understanding how genetic and dietary factors interact throughout the disease course. This study employs a cross-sectional design, limiting our ability to infer causal relationships between variables. Furthermore, participants were recruited from a specific medical center without random sampling, introducing potential selection bias and restricting the generalizability of our findings. To improve generalizability, future research should recruit participants from diverse racial, ethnic, and geographic backgrounds. Such inclusion would allow for the exploration of how population-specific allele frequencies and nutrient profiles influence OA susceptibility, thereby enhancing the relevance of gene-diet findings across global populations.

Second, although we focused on specific genes related to nutrient metabolism and inflammation, there are many other genetic variants that may also contribute to OA risk. For example, variants in genes involved in collagen synthesis, extracellular matrix remodeling, and joint tissue integrity could play a role in OA susceptibility. Future studies should consider including a broader set of genetic markers to provide a more comprehensive understanding of the genetic underpinnings of OA. Moreover, the study did not assess the effect of gene–gene interactions, which could further influence OA risk and severity. The incorporation of whole-genome sequencing or exome sequencing in future research could help identify additional genetic loci involved in OA pathogenesis. Another limitation is the reliance on self-reported dietary intake data. While we attempted to minimize measurement bias through the use of validated dietary assessment tools, self-reported data can be subject to recall bias and inaccuracies in the reporting of nutrient intake. Objective measurements of nutrient levels, such as blood biomarkers, would provide more accurate data on nutrient status and better reflect the actual nutritional intake of participants.

The generalizability of our findings is limited by the demographic characteristics of our study population. The severity of osteoarthritis in all participants was assessed using the Kellgren-Lawrence (K-L) grading system, which classifies OA into five grades:

(1) Grade 0 (None): Definite absence of x-ray changes of osteoarthritis.
(2) Grade 1 (Doubtful): Doubtful joint space narrowing and possible osteophytic lipping.
(3) Grade 2 (Minimal): Definite osteophytes and possible joint space narrowing.
(4) Grade 3 (Moderate): Moderate multiple osteophytes, definite narrowing of joint space, some sclerosis, and possible deformity of bone ends.
(5) Grade 4 (Severe): Large osteophytes, marked narrowing of joint space, severe sclerosis, and definite deformity of bone ends.

The distribution of affected joints was as follows: knee joints accounted for 70%, and hip joints for 30%. OA was categorized based on etiology into primary (idiopathic) and secondary (resulting from other diseases or injuries), with primary OA comprising 85% and secondary OA 15% of cases in this study. Although our sample size of 500 individuals is adequate for the purposes of this analysis, the study cohort was predominantly composed of middle-aged and older adults of a specific geographical region. Future studies should include a more diverse population in terms of age, ethnicity, and geographic location to ensure the results are applicable to a wider population. Additionally, the inclusion of both early-stage and late-stage OA patients would provide a more nuanced understanding of how gene-nutrient interactions influence disease progression.

### 4.5 Suggestions for future research

Future research should aim to address the limitations of this study by incorporating longitudinal study designs to establish causal relationships between genetic factors, nutrient intake, and osteoarthritis risk. Longitudinal studies would allow researchers to track the progression of OA over time and identify whether specific genetic and nutritional factors contribute to the onset or worsening of the disease. Additionally, such studies could examine the temporal dynamics of gene-nutrient interactions, providing deeper insights into the role of diet in modifying genetic risk for OA.

Expanding the genetic analysis to include a wider range of genes involved in OA pathogenesis is also a key area for future research. While our study focused on genes related to omega-3 fatty acid metabolism, vitamin D metabolism, and inflammation, other genes involved in joint integrity, such as those encoding for collagen or matrix metalloproteinases, may also play a significant role in OA risk. A more comprehensive genetic approach, incorporating whole-genome or exome sequencing, could help uncover additional genetic loci associated with OA susceptibility. Additionally, exploring gene–gene interactions could provide a more nuanced understanding of how multiple genetic factors interact to influence OA risk.

To overcome the limitations of self-reported dietary intake data, future studies should incorporate objective measures of nutrient status, such as plasma levels of omega-3 fatty acids, vitamin D, and antioxidants. These biomarkers would provide a more accurate representation of the participants’ nutritional status and allow for a clearer understanding of how nutrient deficiencies or excesses contribute to OA risk in genetically predisposed individuals.

## 5 Conclusion

The association between nutritional factors, genetic predispositions, and the risk of osteoarthritis (OA) is an area of growing interest, particularly as researchers explore how gene–environment interactions contribute to disease onset and progression. This study provides compelling evidence that specific genetic variants related to nutrient metabolism, notably in the FADS1 and VDR (Vitamin D receptor) genes, interact with dietary intake to modulate OA risk. Our findings demonstrate that individuals with certain genetic predispositions may be more vulnerable to the negative effects of nutrient deficiencies, further emphasizing the importance of personalized medicine in managing OA. Specifically, we found that genetic polymorphisms influencing omega-3 fatty acid metabolism, vitamin D receptor function, and inflammatory responses significantly impact OA risk when combined with suboptimal dietary intake of these nutrients. These gene-nutrient interactions not only deepen our understanding of OA pathogenesis but also provide a basis for developing targeted dietary interventions for those at higher genetic risk.

Our study underscores the complexity of OA as a multifactorial disease, wherein both genetic and environmental factors contribute to its development and progression. The integration of genetic data with nutritional assessments has the potential to improve the prediction of OA risk and aid in the design of personalized prevention and treatment strategies. The risk prediction model we developed demonstrated strong predictive power, showing that considering both genetic variants and nutrient intake can offer more accurate risk assessments compared to traditional approaches. This model represents a step forward in identifying individuals at high risk for OA who might benefit from early interventions, such as dietary modifications, to reduce disease burden.

However, despite the promising results, our study also has limitations. The cross-sectional design and the reliance on self-reported dietary data pose challenges to establishing causal relationships. Furthermore, while we focused on specific genes and nutritional factors, there are likely additional genetic variants and dietary components that contribute to OA risk. Future research should aim to include a broader range of genetic markers and a more diverse population to better capture the complexity of OA. Longitudinal studies that track participants over time would provide stronger evidence of causality and help to elucidate the mechanisms by which these genetic-nutrient interactions influence disease onset and progression.

## Data Availability

The original contributions presented in the study are included in the article/supplementary material, further inquiries can be directed to the corresponding author.
